# Retraction Note to: Meta-analysis of the associations between TNF-α or IL-6 gene polymorphisms and susceptibility to lung cancer

**DOI:** 10.1186/s40001-016-0225-x

**Published:** 2016-08-02

**Authors:** Wei Zhou, Shuxiang Zhang, Yingchun Hu, Jianrong Na, Na Wang, Xuan Ma, Lizhi Yuan, Fanzhen Meng

**Affiliations:** Department of Respiration, General Hospital of Ningxia Medical University, Shengli Street, Yinchuan, 750004 China

## Retraction to: European Journal of Medical Research (2015) 20:28 DOI 10.1186/s40001-015-0113-9

The Publisher and Editor are retracting this article [1] because the authorship of the article cannot be confirmed and the peer review process was compromised. As a result, the scientific integrity of the article cannot be guaranteed. The institution has informed BioMed Central that the authors involved a third party to assist with copyediting and submission to the journal. Fanzhen Meng did not respond to our correspondence about this retraction. All other authors support the retraction of this article.
